# Successful Management of Immune Thrombocytopenia Presenting with Lethal Alveolar Hemorrhage

**DOI:** 10.1155/2019/5170282

**Published:** 2019-06-10

**Authors:** Keiki Nagaharu, Masahiro Masuya, Keiki Kawakami, Naoyuki Katayama

**Affiliations:** ^1^Department of Hematology and Oncology, Mie University Graduate School of Medicine, Edobashi 2-174, Tsu, Mie Prefecture 514-8507, Japan; ^2^Department of Hematology and Oncology, Suzuka General Hospital, Yamanohana 1275-53, Suzuka, Mie Prefecture 513-8630, Japan

## Abstract

Immune thrombocytopenic purpura (ITP) typically presents with bleeding due to immunologic thrombocytopenia. Severe hemorrhage due to ITP is sometimes lethal, and the urgent recovery of platelets is necessary. In addition to conventional therapeutic strategies, romiplostim shows promising efficacy for chronic ITP. However, there is little evidence for the utilization of this treatment for acute ITP or acute exacerbation of chronic ITP. We report the case details of three elderly ITP patients presenting with lethal diffuse alveolar hemorrhage. These patients had the following underlying pulmonary diseases: case 1, nontuberculous mycobacterial infection and sarcoidosis; case 2, cryptogenic organizing pneumonia; case 3, pulmonary emphysema. These patients recovered following treatment with romiplostim at a higher dose (10 *μ*g/kg), in addition to conventional therapies including corticosteroids and high-dose intravenous immunoglobulin. In summary, the addition of romiplostim resulted in earlier recovery of thrombocytopenia than has been previously reported. Our three cases suggest that early romiplostim at a higher dose could be an efficacious therapeutic option for acute ITP patients with severe lethal bleeding.

## 1. Introduction

Immune thrombocytopenic purpura (ITP) is a hematological disorder characterized by thrombocytopenia due to immunological mechanisms. Lethal bleeding symptoms (e.g., intracranial, gastrointestinal, and diffuse alveolar hemorrhage (DAH)) have been reported as severe complications in ITP [1]. Standard first-line therapy for ITP is corticosteroids, with or without high-dose intravenous immunoglobulin (IVIG). In cases refractory to these therapies, splenectomy or treatment with rituximab is recommended [2–4]. Recently, romiplostim has been reported as an additional strategy [5]. In chronic ITP, weekly subcutaneous administration of romiplostim increased the platelet count without significant adverse effects [6, 7]. However, few reports have focused on early care with romiplostim for newly diagnosed patients or cases of acute exacerbation of ITP [8, 9]. We report three elderly ITP patients presenting with lethal DAH. These cases were successfully treated with romiplostim at a higher dose, in addition to conventional therapies. Through our cases, we suggest the efficacy of romiplostim at a higher dose against lethal hemorrhage.

## 2. Case Presentation

### 2.1. Case 1

A 65-year-old male was admitted to our hospital because of ecchymosis on both lower extremities. Three years before admission, he was diagnosed with ITP by laboratory tests, including antibodies against platelet glycoprotein (GP) IIb/IIIa and GP IV, and bone marrow aspiration. He had been treated successfully with corticosteroids. History included nontuberculosis mycobacterial infection. Previous treatment included prednisolone (PSL; 5 mg/day), clarithromycin, rifampicin, and ethambutol hydrochloride. Two weeks before admission, routine laboratory examination showed normal platelet counts (PC; 185 × 10^9^/l).

On admission, laboratory findings showed a PC of 3.0 × 10^9^/l. Biochemical parameters and coagulation values were within the normal limit. Antibodies against *Helicobacter pylori*, hepatitis C virus (HCV), hepatitis B virus (HBV), and human immunodeficiency virus (HIV) were negative. We diagnosed him with acute exacerbation of chronic ITP. The clinical course is shown in Supplementary Figure 1.

He was treated immediately with high-dose IVIG, PSL (40 mg/day, p.o.), and romiplostim (1 *µ*g/kg). During the next four days, he developed respiratory failure; PaO_2_/FiO_2_ ratio was approximately 250. On the fourth day of hospitalization, computed tomography (CT) revealed ground-glass opacities (GGOs) with marginal infiltration in both lung fields, on the basis of which a diagnosis of alveolar hemorrhage was made. His dyspnea worsened gradually, and noninvasive positive pressure ventilation (NPPV) was initiated.

On the 11^th^ day of hospitalization, a higher dose of romiplostim (10 *µ*g/kg) was initiated together with pulsed doses of methyl-PSL (1000 mg/day for 3 days) and a second cycle of IVIG. His PC recovered by the 21^st^ day of hospitalization, and he was discharged without any complications. The patient's PC has remained normal whilst being treated with 12.5 mg of PSL daily for 12 months, without recurrence of alveolar hemorrhage.

### 2.2. Case 2

A 71-year-old male was referred to our hospital because of gingival bleeding, hemoptysis, and dyspnea. His past medical history included acute HBV infection during the pediatric period, which resolved without any prolonged hepatic disorders. Three months prior to admission, chest radiography showed a consolidation in the right lung field. Two days before admission, he underwent transbronchial lung biopsy and was diagnosed with cryptogenic organizing pneumonia.

The patient's PC was low (6.0 × 10^9^/l) at admission, despite a normal PC (224 × 10^9^/l) at 12 days before admission. Platelet-associated IgG increased to 81.7 ng/10^7^ cells, and tests for antibodies against GP IIb/IIIa and GP Ia/IIa were positive. Serological tests for *H. pylori*, HCV, and, HIV were negative but positive for anti-HBs antibody, without viremia. Bone marrow examination showed an increased number of megakaryocytes, without apparent malignancy. CT at admission revealed pulmonary bilateral GGOs. The patient was diagnosed as having acute ITP with alveolar hemorrhage.

High-dose IVIG was started with PSL (0.5 mg/kg, p.o.); however, he developed dyspnea on the second day of hospitalization (Supplementary Figure 1). Given the risk for respiratory failure, a higher dose of romiplostim (10 *μ*g/kg/w) was initiated. Nine days after admission, his PC increased to 9.0 × 10^9^/l and respiratory symptoms resolved. On the 30^th^ day after admission, he was discharged without any complications.

### 2.3. Case 3

A 71-year-old male was admitted because of dyspnea and hemoptysis. He had a history of Stevens–Johnson syndrome due to salazosulfapyridine and pulmonary emphysema. On admission, he developed severe respiratory failure (P/F ratio: 100) and NPPV support was initiated. His PC was low (2.0 × 10^9^/*µ*l) at admission but had been normal (158 × 10^9^/l) two months before. Laboratory tests showed high PA-IgG (605.8 ng/10^7^ cells), and serological tests for *H. pylori*, HCV, HBV, and HIV were negative. Bone marrow examination revealed an increase in the number of megakaryocytes without malignancies. CT on the day of admission revealed GGOs with no evidence of infection. He was diagnosed as having acute ITP with alveolar hemorrhage.

The patient was treated with methyl-PSL pulse therapy (1000 mg daily for 5 days) and IVIG (Supplemental Figure 1). Rapid progression of severe respiratory failure occurred over the next two days; hence, we added romiplostim at a higher dose (10 *μ*g/kg weekly). Four days after initiating treatment, his PC increased to 25 × 10^9^/l and hemoptysis and dyspnea resolved. Despite tapering of the PSL dose, there was no recurrence of thrombocytopenia.

## 3. Discussion

The causes of DAH are heterogeneous: vasculitis, drug-induced lung injury, stem cell transplantation, infection, trauma, coagulopathy, and thrombocytopenia [10, 11]. The prognosis of thrombocytopenia-related DAH is especially poor [12]. ITP patients with DAH are rare although are described in several case reports [13–17]. Although these cases have no apparent relationship with pulmonary diseases, our three patients had underlying pulmonary diseases, in addition to thrombocytopenia. Clinicians should consider the importance of DAH in ITP with underlying lung diseases.

IVIG and corticosteroids are reported to result in recovery in approximately 2 days and 2–5 days, respectively [1]. Alternative strategies including splenectomy and/or thrombopoietin receptor agonists should be considered in refractory cases. The usual initiation dose of romiplostim is 1.0 *µ*g/kg, and dose adjustment is necessary to achieve PC responses. The standard initiation dose is not sufficient in many cases. Indeed, Shirasugi et al. reported that treatment with an initial dose of 3 to 6 *µ*g/kg promotes more rapid platelet recovery compared with that of 1.0 *µ*g/kg in chronic ITP [18]. In addition, Contis et al. showed that higher doses (6–9 *µ*g/kg) of romiplostim might be needed in patients with severe acute or persistent ITP, who are refractory to conventional therapies [8]. These findings indicated that the higher dose is effective for lethal cases.

**Figure 1:**
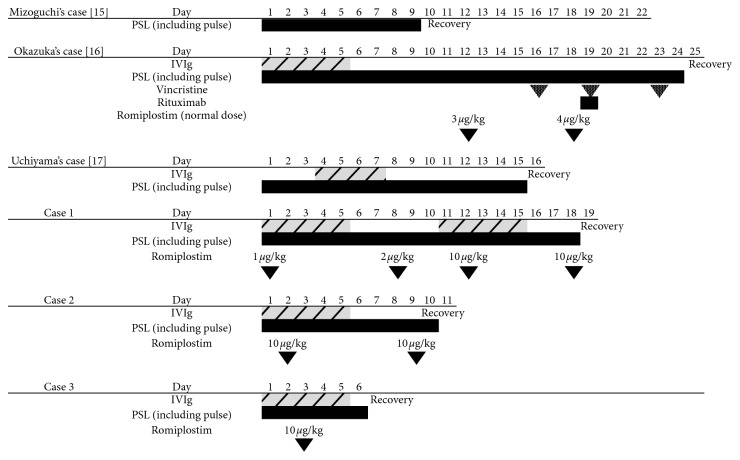
Literature review of the cases with DAH due to ITP. Recovery is defined as a platelet count of more than 100 × 10^9^/l.

Our cases showed a more rapid response, compared with previous cases with available clinical data [15–17] (Figure 1). Despite having a much higher platelet count (600 to 1000 × 10^9^/l) after treatment (Supplementary Figure 1), no vascular thrombosis was observed in all cases. These cases indicated that early and higher doses of romiplostim can be effective for ITP with lethal hemorrhage. Further investigations will be needed to establish the appropriate dose of romiplostim for patients with ITP presenting with lethal hemorrhage.

## Supplementary Materials

Supplementary Materials Supplementary Figure 1 shows clinical course of our 3 cases.
