# Supplementary material for: Successful Management of Immune Thrombocytopenia Presenting with Lethal Alveolar Hemorrhage
Source: Case Rep Hematol. 2019 Jun 10;2019:5170282. doi: 10.1155/2019/5170282 (PMC6590510; doi:10.1155/2019/5170282)
Supplement: Supplementary Materials — Supplementary Figure 1 shows clinical course of our 3 cases. [file 5170282.f1.pptx]

## Slide 1
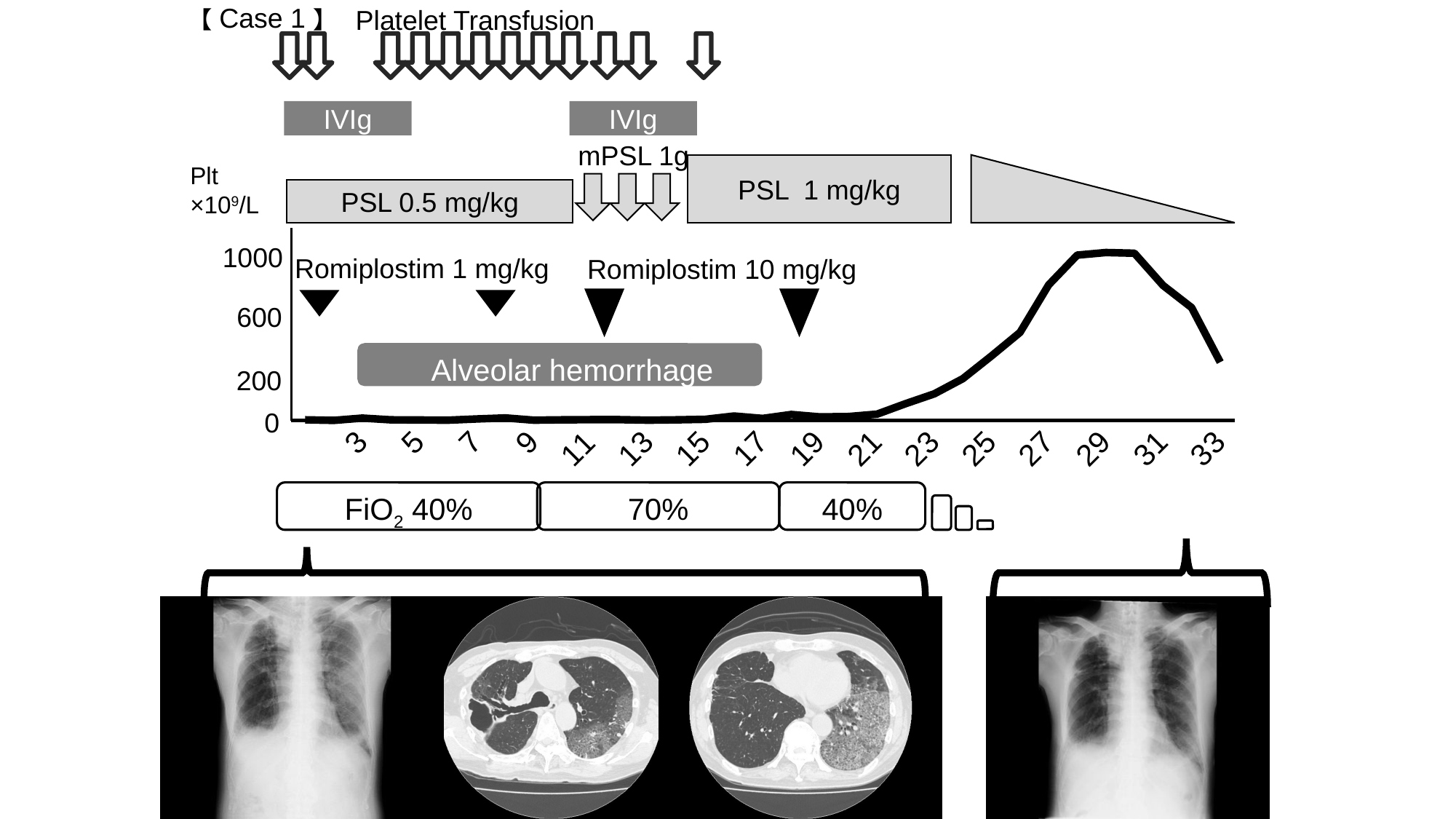

【Case 1】
Platelet Transfusion
IVIg
IVIg
mPSL 1g
Plt
×109/L
PSL 1 mg/kg
PSL 0.5 mg/kg
### Chart
| Category | Platelet count |
|---|---|
| day1 | 0.30000000000000027 |
| 2 | 0.0 |
| 3 | 1.3 |
| 4 | 0.30000000000000027 |
| 5 | 0.2 |
| 6 | 0.1 |
| 7 | 0.8 |
| 8 | 1.4 |
| 9 | 0.1 |
| 10 | 0.30000000000000027 |
| 11 | 0.4 |
| 12 | 0.4 |
| 13 | 0.1 |
| 14 | 0.30000000000000027 |
| 15 | 0.6000000000000005 |
| 16 | 2.5 |
| 17 | 1.1 |
| 18 | 3.4 |
| 19 | 2.0 |
| 20 | 2.2 |
| 21 | 3.6 |
| 22 | 9.700000000000001 |
| 23 | 15.4 |
| 24 | 24.2 |
| 25 | 37.4 |
| 26 | 51.1 |
| 27 | 78.7 |
| 28 | 96.0 |
| 29 | 97.5 |
| 30 | 97.1 |
| 31 | 78.4 |
| 32 | 65.5 |
| 33 | 33.800000000000004 |
1000
Romiplostim 1 mg/kg
Romiplostim 10 mg/kg
600
 Alveolar hemorrhage
200
0
FiO2 40%
70%
40%

## Slide 2
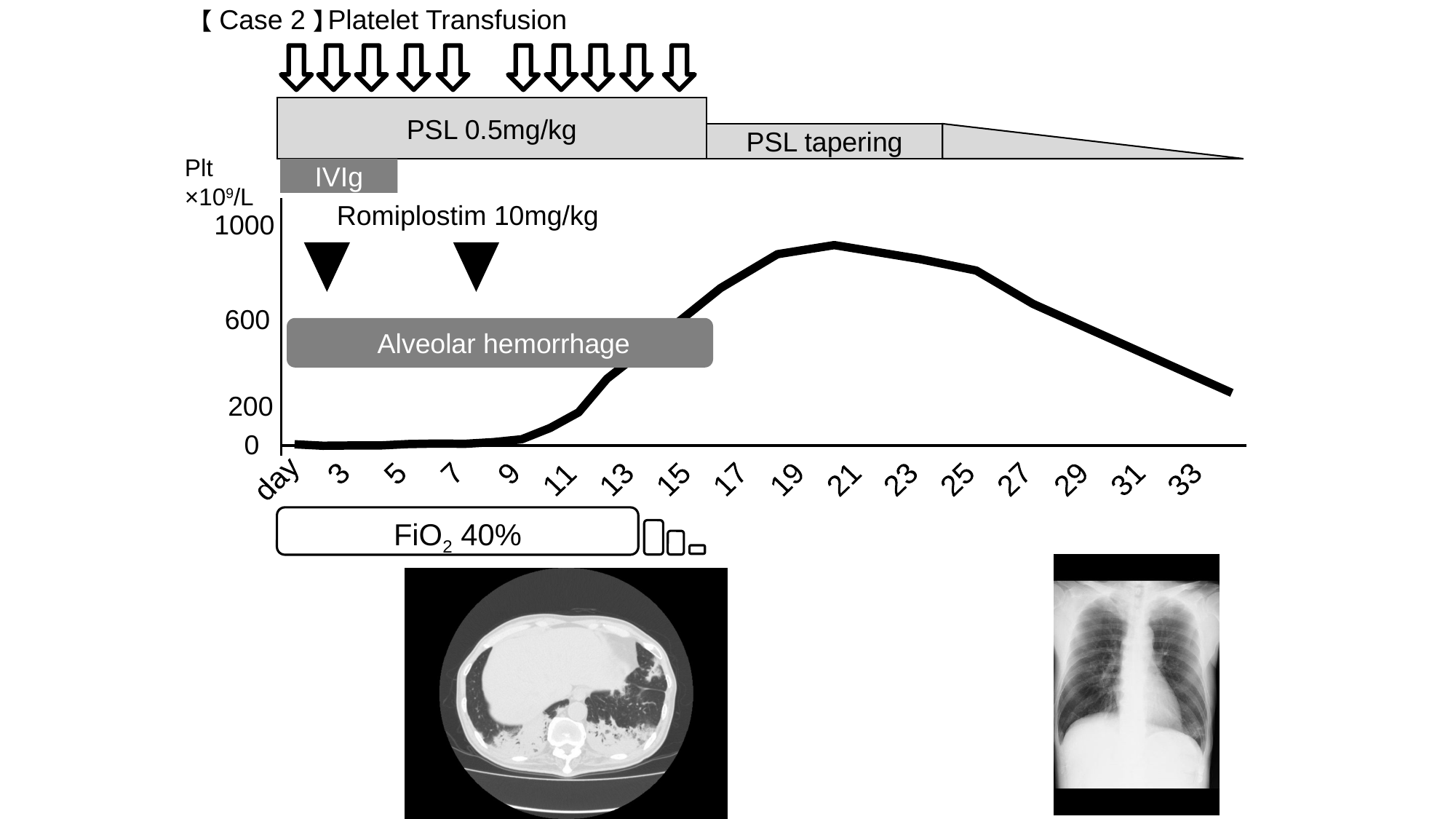

【Case 2】
Platelet Transfusion
PSL 0.5mg/kg
PSL tapering
Plt
×109/L
IVIg
Romiplostim 10mg/kg
1000
### Chart
| Category | Platelet count |
|---|---|
| day1 | 0.6000000000000005 |
| 2 | 0.0 |
| 3 | 0.1 |
| 4 | 0.1 |
| 5 | 0.7000000000000005 |
| 6 | 0.9 |
| 7 | 0.8 |
| 8 | 1.5 |
| 9 | 2.7 |
| 10 | 7.4 |
| 11 | 13.9 |
| 12 | 27.9 |
| 13 | 37.2 |
| 14 | None |
| 15 | None |
| 16 | 65.7 |
| 17 | None |
| 18 | 79.8 |
| 19 | None |
| 20 | 83.6 |
| 21 | None |
| 22 | None |
| 23 | 77.8 |
| 24 | None |
| 25 | 72.98 |
| 26 | None |
| 27 | 59.1 |
| 28 | None |
| 29 | None |
| 30 | None |
| 31 | None |
| 32 | None |
| 33 | None |
| 34 | 22.0 |
600
 Alveolar hemorrhage
200
0
FiO2 40%

## Slide 3
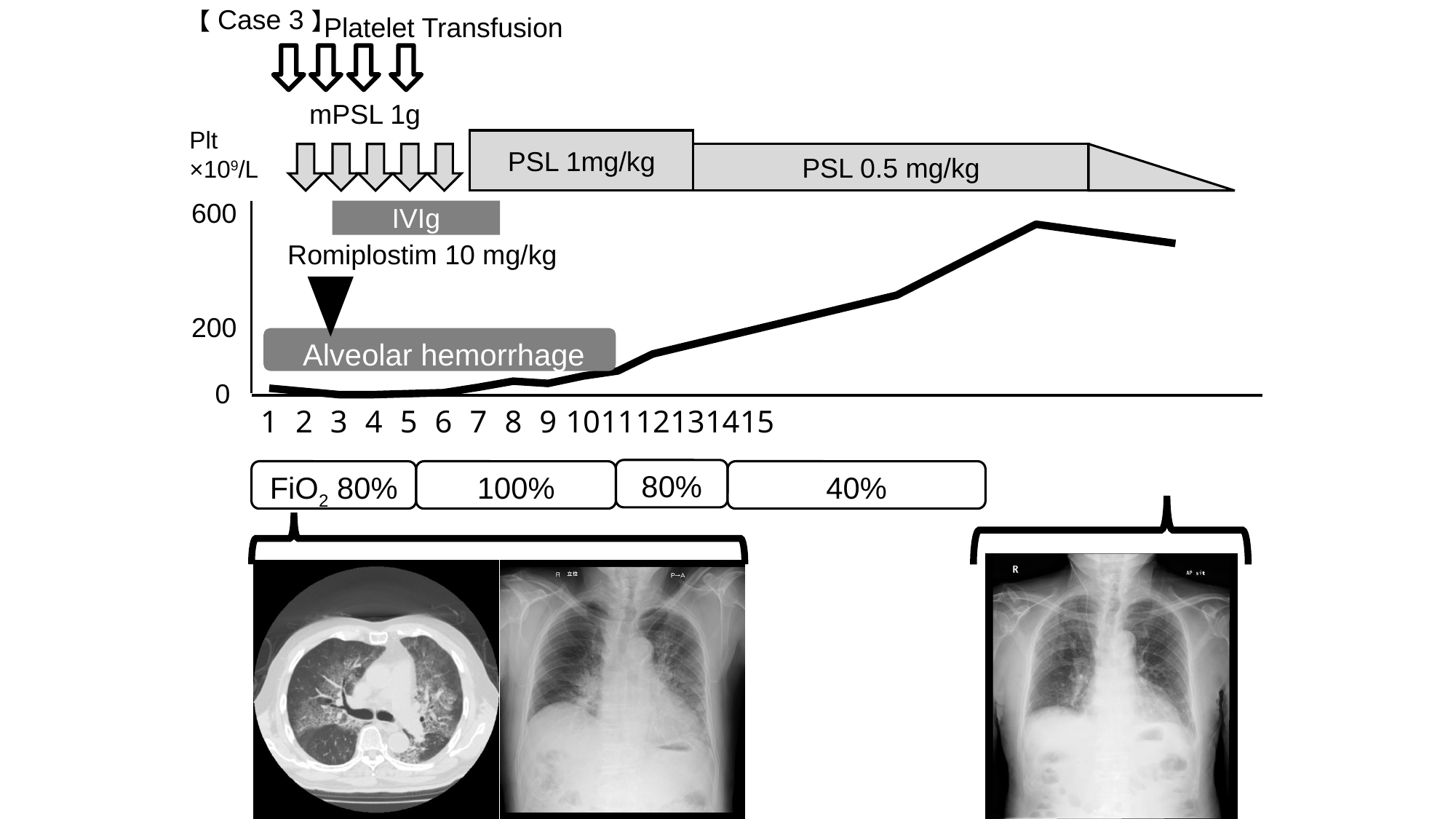

【Case 3】
Platelet Transfusion
mPSL 1g
Plt
×109/L
PSL 1mg/kg
PSL 0.5 mg/kg
### Chart
| Category | Plt |
|---|---|600
IVIg
Romiplostim 10 mg/kg
200
 Alveolar hemorrhage
0
80%
FiO2 80%
100%
40%
